# [Retracted] Correlative study on the JAK‑STAT/PSMβ3 signal transduction pathway in asthenozoospermia

**DOI:** 10.3892/etm.2025.12831

**Published:** 2025-02-24

**Authors:** Junguo Li, Li Zhang, Bing Li

Exp Ther Med 13:127–130, 2017; DOI: 10.3892/etm.2016.3959

Following the publication of the above paper, a concerned reader drew to the Editor's attention that, regarding the PCR gel portrayed in Fig. 1A on p. 128, unexpected areas of similarity (both bands and the background) were noted; in addition, both control bands and bands related to the proteins of interest had apparently been duplicated in the western blot shown in Fig. 2A, where the results from different experiments were intended to have been shown.

Following an internal enquiry, the Editor of *Experimental and Therapeutic Medicine* has been able to verify the claims made by the interested reader; therefore, in view of the abovementioned anomalies that were identified, and owing to an overall lack of confidence in the presented data, the Editor has decided that the paper should be retracted from the Journal. The authors were contacted regarding these matters, but we received no reply from them. The Editor apologizes to the readership of the Journal for any inconvenience caused.

